# Dynamics of Agriculture 4.0 Technology Adoption in the Agri-Food System: Insights from an Exploratory Study in Rio Grande do Sul—Brazil

**DOI:** 10.3390/foods15111892

**Published:** 2026-05-27

**Authors:** Franco da Silveira, Dheeraj Bharti, Irem Kılınç, Danielle Elis Garcia Furuya, Everton Castelão Tetila, Carlos Parra-López, Édson Luis Bolfe, Thiago Teixeira Santos, Jayme Garcia Arnal Barbedo

**Affiliations:** 1 Brazilian Agricultural Research Corporation, Embrapa Digital Agriculture, Av. André Tosello, 209-C.P. 6041, Campinas 13083-886, SP, Brazil; dheeraj.nitb@gmail.com (D.B.); danielle.furuya@colaborador.embrapa.br (D.E.G.F.); edson.bolfe@embrapa.br (É.L.B.); thiago.santos@embrapa.br (T.T.S.); jayme.barbedo@embrapa.br (J.G.A.B.); 2 Fish Processing Technology Department, Fisheries Faculty, Katip Çelebi University, 35640 Çiğli, Izmir, Turkey; ilincirem75@gmail.com; 3 Federal University of Grande Dourados, Rodovia Dourados/Itahum, Km 12, Cidade Universitária, Dourados 79804-970, Brazil; evertontetila@ufgd.edu.br; 4 Department of Agrifood System Economics, Institute of Agricultural and Fisheries Research and Training (IFAPA), Camino de Purchil, s/n, 18004 Granada, Spain; carlos.parra@juntadeandalucia.es

**Keywords:** food security, digital agriculture, precision agriculture, innovation adoption, diffusion of innovations, barriers, co-occurrence network, agri-food system

## Abstract

Despite the growing relevance of Agriculture 4.0 technologies for enhancing productivity, decision-making, and sustainability in agri-food systems, their adoption remains uneven in developing-country contexts. This study aims to analyze the perceived severity and co-occurrence structure of barriers to Agriculture 4.0 adoption in the agri-food system of Rio Grande do Sul (RS), Brazil, using an exploratory quantitative design grounded in a barrier co-occurrence perspective rather than a causal or actor-centered network interpretation. An online survey conducted in 2024 with farmers in RS evaluated 25 literature-validated barriers spanning technological, economic, political, social, and environmental dimensions. The analysis combined a Barrier Severity Index (BSI), reliability testing, Principal Component Analysis (PCA), K-means clustering, ANOVA by farm size, and proximity-based co-occurrence networks constructed from highly rated barriers. The results show that economic barriers remain the most severe overall, particularly the lack of affordable solutions, high maintenance costs, and limited infrastructure. At the same time, farm-size-stratified networks reveal distinct association structures: small farms display a more segmented pattern linking affordability and technical access to institutional and capability constraints; medium farms show the most globally integrated co-occurrence structure; and large farms exhibit a dense but more differentiated configuration combining cost, interoperability, skills, and governance-related barriers. These findings are interpreted descriptively, as the networks capture patterns of co-reporting rather than causal interdependence. The study contributes a network-analytic representation of perceived barrier configurations and highlights the need for scale-sensitive policy mixes that address bundles of constraints rather than isolated obstacles.

## 1. Introduction

Over the past decades, the demands placed on agri-food systems have become one of the major global concerns. The world population is expected to increase significantly by 2050, which is likely to result in a substantial rise in the demand for sustainably produced food [1,2]. This scenario imposes the need to expand agricultural productive capacity while intensifying the efficient and responsible use of natural resources [3]. However, persistent environmental challenges—such as the reduction in arable land, climate change, soil degradation, and water resource limitations—continue to represent significant obstacles for contemporary agricultural production [4]. Taken together, these pressures highlight the urgency of structural and systemic transformations in agricultural practices capable of reconciling productivity, quality, and sustainability within agri-food systems [1,2,3,4].

In this context, Agriculture 4.0, also known as the Fourth Agricultural Revolution, emerges as a socio-technical paradigm grounded in the systemic integration of emerging technologies into the agri-food system [5]. Unlike previous approaches, Agriculture 4.0 is not limited to the isolated adoption of innovations but involves the articulation of an interconnected technological ecosystem, including robotics, cloud computing, big data analytics, Artificial Intelligence (AI), the Internet of Things (IoT), Unmanned Aerial Vehicles (UAVs or drones), blockchain, Digital Twins (DTs), Machine Learning (ML), Remote Sensing (RS), and intelligent sensor networks. These technologies operate in an integrated manner across agricultural and food production chains, enhancing the capacity for monitoring, automation, and management of production processes [5,6].

Thus, Agriculture 4.0 refers to the strategic application of these emerging technologies within the agri-food system to address growing challenges related to resource-use efficiency, environmental sustainability, and the operational complexity of the system itself [5,7]. The generation and processing of high-resolution, real-time data contribute to improved decision-making and operational efficiency in increasingly dynamic and uncertain production environments [7,8]. As a result, these technologies enable the optimization of critical inputs such as water, fertilizers, and energy, the reduction in environmental impacts, and the increase in agricultural productivity [9], as well as reducing the use of chemical inputs and improving soil health [10,11].

Brazil occupies a strategic position in the global agri-food system, both due to its high production volume and the diversity of its production systems, which gives the country significant potential to advance in the adoption of technologies associated with Agriculture 4.0 [12,13]. Despite this potential, the incorporation of these technologies occurs unevenly across the national territory, reflecting structural asymmetries among regions, production systems, and farmer profiles. In particular, farm size emerges as a critical structural dimension shaping access to resources, infrastructure, and institutional support. Small and medium-sized farms tend to face greater difficulties in this process, which go beyond strictly technical issues and involve economic constraints, infrastructure deficiencies, institutional gaps, and sociocultural barriers [14,15,16].

Empirical evidence in the Brazilian agri-food system indicates that Technology Adopter Farmers (TAFs) and Non-Technology Adopter Farmers (NTAFs) face distinct, although interrelated, sets of barriers to Agriculture 4.0 adoption. Among TAFs, challenges are less associated with initial access to technologies and more related to their operation, adaptation, and effective integration into the agri-food system. Issues related to available infrastructure, technological complexity, and the suitability of solutions to local farm realities limit the full exploitation of these innovations’ potential. In addition, economic constraints, as well as political-institutional factors—including the absence of farmer-centered approaches—negatively influence the consolidation of Agriculture 4.0. Social factors, especially educational gaps, and environmental challenges related to data reliability and technological sensitivity to climatic conditions also continue to compromise expected outcomes, even among farmers who have already adopted Agriculture 4.0 technologies [14]. For NTAFs, barriers are predominantly structural and systemic, directly affecting the initial decision to adopt Agriculture 4.0 technologies. The lack of basic infrastructure, high costs of acquisition, implementation, and maintenance, as well as the scarcity of affordable solutions, constitute central obstacles in this process. These constraints are often reinforced by farm size, which limits investment capacity and bargaining power. Such barriers are further intensified by limitations in the political-institutional environment, including weak targeted public policies, low investment in research and development, and the absence of clear national strategies for implementing Agriculture 4.0. Additionally, educational challenges and limited access to reliable rural data contribute to perceptions of risk, uncertainty, and technological exclusion among these farmers [14].

More broadly, these barriers not only restrict individual technology adoption but also reinforce persistent inequalities in access to and implementation of Agriculture 4.0 across different regions and farm sizes, thereby deepening structural asymmetries within the Brazilian agri-food system [15,17,18]. Although the potential of emerging technologies is widely recognized, empirical studies that deeply investigate how Brazilian farmers perceive, interpret, and experience these obstacles within their specific social, institutional, and structural contexts remain scarce [18]. Recent evidence suggests that overcoming these limitations—especially in organic and agroecological systems—is strongly associated with the strengthening of collaborative networks, data sharing, knowledge exchange, and effective information governance mechanisms, highlighting the relational dimension of technological adoption in rural areas [19].

Although the literature has identified many of these barriers, empirical studies often still treat them as isolated variables or as independent items in rankings and regression analyses [15,17]. Such approaches are useful for establishing prevalence or severity but do not fully capture how barriers are jointly perceived by respondents. In practice, farmers rarely face one challenge at a time; instead, they often report sets of difficulties that span technical, economic, social, and institutional dimensions. This point is analytically relevant because when a farmer states that Agriculture 4.0 technologies are difficult to adopt, this perception may involve not only costs but also connectivity, lack of skills, absence of locally appropriate solutions, and insufficient institutional support [17,18]. A variable-by-variable analysis may identify which barriers are important on average but may fail to capture how these constraints are associated in farmers’ own evaluations.

For this reason, the present study adopts a network-based analytical representation of perceived barriers. More specifically, it constructs a proximity-based barrier co-occurrence network, in which nodes represent predefined barriers from the literature, and edges represent the tendency for these barriers to be jointly rated as important by the same respondents. This constitutes a descriptive, variable-centered use of network analysis. The study does not measure interpersonal ties, actor relations, information flows, or social-network diffusion among farmers, firms, extension agents, or institutions. The analytical purpose is therefore descriptive co-occurrence mapping of perceived barriers, not classical social network analysis.

The study does not examine social ties among farmers, extension agents, firms, or institutions, nor does it aim to test social network theory in the classical actor-relational sense [20,21,22,23,24,25,26,27]. Instead, it uses network visualization and proximity analysis as exploratory tools to represent patterns of association among barrier perceptions. In this sense, the approach is closer to co-occurrence mapping and proximity-based relational analysis than to studies of diffusion through interpersonal networks.

This approach is particularly suitable for an exploratory survey such as the present one. Since the instrument is based on 25 barriers previously identified and validated in the literature [5,18], the objective is not to induce new barriers from open-ended narratives but to understand how established barriers vary in terms of perceived severity and how they are associated across farm-size groups [28,29,30].

Thus, the contribution of the study is twofold. Empirically, it provides evidence on the severity and configuration of Agriculture 4.0 barriers in an important agricultural region of Brazil. Analytically, it combines severity measures, multivariate exploration, inferential comparisons by farm size, and descriptive co-occurrence networks, moving beyond single-barrier interpretations. This study aims to analyze the perceived severity of barriers to Agriculture 4.0 adoption among farmers and to examine how these barriers are associated with each other across different farm-size groups. The central research question is therefore formulated as follows: **Which barriers to the adoption of Agriculture 4.0 technologies are perceived as most severe by farmers in Rio Grande do Sul, Brazil, and how are the most highly rated barriers associated with one another across different farm-size groups?**

The study relies on a non-probabilistic online survey and should be interpreted as an exploratory case study rather than a statistically representative portrait of Brazilian agriculture. Even so, it offers a useful analytical lens for identifying central barriers, recurring co-occurrence patterns, and structural differences in perceived constraints according to production scale. From a practical standpoint, this approach can help policymakers, cooperatives, rural extension services, and technology providers avoid one-dimensional interventions. If barriers tend to be perceived in bundles, effective responses may also need to be designed as integrated interventions combining economic affordability, infrastructure, capacity building, and institutional support.

The remainder of this article is organized as follows. Section 2 presents the background on the barriers analyzed. Section 3 describes the methodological approach. Section 4 presents the results. Section 5 discusses the findings, and Section 6 concludes with implications and directions for future research.

## 2. Background

The adoption of emerging technologies associated with Agriculture 4.0 has been widely recognized as a complex and multifaceted process that goes beyond individual farmers’ decisions or the mere availability of technological solutions in the market [31]. Within the context of agri-food systems, the adoption of emerging technologies is shaped by a broad set of barriers that operate simultaneously, cumulatively, and interdependently, encompassing technological, economic, political, social, and environmental dimensions [5,32]. These barriers tend to manifest heterogeneously across regions, production types, and farmer profiles, reflecting structural inequalities historically present in rural areas, particularly in developing countries such as Brazil [14,15,18,33].

Several studies indicate that these barriers do not act in isolation but often reinforce one another, creating lock-in dynamics that hinder both the initial adoption and the sustained use of Agriculture 4.0 technologies [34,35]. Infrastructure constraints, for instance, can exacerbate economic barriers; institutional weaknesses can undermine technical training; and educational gaps can intensify perceptions of risk and uncertainty associated with emerging technologies [36,37]. For this reason, the analytical challenge is not simply to catalogue barriers, but to understand how different kinds of barriers appear together in farmers’ evaluations. The literature has long acknowledged that adoption involves multiple dimensions, yet empirical analyses still frequently privilege isolated rankings or single-variable treatments [38,39]. A co-occurrence perspective complements those approaches by asking which barriers tend to be jointly perceived as important.

The Agriculture 4.0 literature identifies a recurring set of 25 barriers to the adoption of emerging technologies in the agri-food system, which can be organized into five major analytical dimensions: technological, economic, political, social, and environmental [5,18]. These categories organize the survey instrument used in this study and provide the conceptual foundation for the subsequent severity, multivariate, and co-occurrence analyses. Furthermore, these barriers represent structural and operational constraints that shape technology adoption decisions across different production contexts. Table 1 presents the systematization of these barriers.

The barriers listed in Table 1 were not created ad hoc for this study. Rather, they were derived from prior literature syntheses and subsequently validated in empirical studies on the adoption of Agriculture 4.0 technologies [5,18]. Their use in this study therefore supports conceptual continuity while allowing the analysis to focus on how these barriers are distributed and associated within a specific regional sample.

Many of these barriers are embedded in the everyday functioning of the agri-food system, becoming normalized within production routines, continuous adaptations to climatic and market conditions, and persistent institutional and infrastructural constraints. As farmers primarily engage with immediate, practical, and operational challenges, the effects of these barriers are often experienced without being explicitly recognized as structural or systemic constraints. Consequently, difficulties related to technology adoption tend to be interpreted at an individual or farm-management level, while broader systemic limitations remain largely invisible in farmers’ day-to-day experiences.

This perception gap is particularly pronounced in contexts such as Brazil, where historical structural inequalities, regional heterogeneity, and disparities in access to key resources—such as credit, technical assistance, digital connectivity, and agricultural equipment—directly affect daily production activities and condition the adoption of Agriculture 4.0 technologies [15,18,40]. In this sense, Table 1 plays a central role by providing a systemic perspective on adoption barriers, making explicit how a set of interconnected technological, economic, political, social, and environmental constraints jointly shape the functioning of the modern agri-food system. By reframing these barriers as systemic rather than individual obstacles, the table offers a more robust analytical foundation for understanding the conditions under which farmers adopt—or fail to adopt—Agriculture 4.0 technologies. The analytical value of this study lies precisely in making these patterns of association visible without overinterpreting them as causal mechanisms.

## 3. Methodology

### 3.1. Study Context

The study was conducted in the context of the agri-food system of Rio Grande do Sul (RS), Brazil—a region of great economic relevance for the country [41]. RS is characterized by a diversity of crops, including rice, wheat, soybeans, corn, and fruits such as grapes, apples, and oranges, as well as meat production (beef, pork, and poultry).

The agricultural structure is heterogeneous, mainly composed of small rural properties, complemented by medium and large farms that occupy a significant portion of the cultivated area [42,43]. RS has approximately 365,000 agricultural establishments distributed across 21.7 million hectares, with a predominance of diversified and multifunctional family farmers, while medium and large farms tend to specialize in monoculture [42,44]. Additionally, the state has relatively high access to cooperatives, technical assistance, and agricultural infrastructure, factors that favor the introduction of emerging technologies and make RS a representative environment to study barriers to the adoption of Agriculture 4.0 [45,46].

### 3.2. Research Design

This study adopts an exploratory quantitative approach, investigating the adoption of emerging technologies associated with Agriculture 4.0, a phenomenon still in the process of consolidation in the Brazilian agri-food system [14,15]. Although international literature has already identified several barriers to the adoption of these technologies, empirical evidence analyzing the relational structure of these barriers from the farmers’ perspective remains scarce, especially in specific regional contexts [18,47,48]. In this sense, the exploratory nature of the research does not stem from the absence of previous studies, but from the need to go beyond the isolated identification or measurement of barriers, seeking to understand how these constraints are organized, interact, and mutually reinforce each other [37], toward a descriptive mapping of co-occurrence patterns across farm sizes. The goal is therefore not to test causal hypotheses, but to identify robust empirical regularities and analytically useful association structures.

### 3.3. Data Collection Procedures

The research instrument consisted of a structured, closed-ended questionnaire administered online (survey), developed based on a set of barriers previously identified by Da Silveira et al. [5] and later validated by Da Silveira et al. [18] in the field of Agriculture 4.0 (see Table 1). The adoption of this consolidated theoretical-empirical framework ensured that the barriers analyzed possess conceptual consistency, empirical relevance, and alignment with the scientific debate, avoiding the introduction of poorly defined or excessively context-specific categories [16,49].

The choice of a closed-ended questionnaire rather than open-ended questions is justified by several factors in this study. First, Agriculture 4.0 involves emerging and complex technologies—e.g., the Internet of Things (IoT), Artificial Intelligence (AI), and drones—whose technical knowledge varies significantly among farmers, who exhibit different levels of familiarity and experience with these technologies [14,34,50,51]. In this context, open-ended questions tend to highlight knowledge limitations rather than structured perceptions of the phenomenon, resulting in fragmented, generic, or difficult-to-compare responses, as occurs among Brazilian farmers.

Second, the objective of the study is not to identify new barriers inductively, but to assess the perceived severity of barriers already established in the literature and to examine how the most highly rated barriers tend to co-occur in participants’ responses. Standardized response options also enable the application of the Barrier Severity Index (BSI), reliability measures, Principal Component Analysis (PCA), cluster analysis, ANOVA, and the construction of proximity-based networks in an internally consistent manner [52,53,54]. In this way, the instrument not only captures structured perceptions but also provides a relational diagnosis of the barriers, revealing strategic leverage points for public policies and technology diffusion practices, especially in contexts of inequality and technological exclusion [27,55].

At the same time, the use of a structured questionnaire imposes an important interpretative limitation: the resulting analysis captures perceived configurations of barriers within the research instrument, rather than the full experiential richness of farmers’ technological adoption trajectories. For this reason, the results should be interpreted as a structured exploratory diagnosis, rather than as an exhaustive ethnographic or causal analysis.

The questionnaire developed for this study was structured into three sections. The first section presented the research objective, the researchers and their institutions, contact information, and guarantees of anonymity and confidentiality. The second section addressed characteristics of farmers and their farms, including gender, education, location, cultivated area, type of crop, and years of experience, providing context for the analysis. The third section focused on the 25 barriers to technology adoption, organized into five categories—technological, economic, political, social, and environmental—each accompanied by a brief explanation to facilitate understanding. The barriers were assessed using a five-point Likert scale, ranging from “not important at all” to “very important”.

### 3.4. Sampling Strategy

The study adopted a non-probabilistic exploratory sampling, appropriate for initial studies on complex and under-documented phenomena such as the adoption of Agriculture 4.0 technologies in the Brazilian agri-food system, with a regional focus on RS. This type of sampling allows for obtaining preliminary insights from accessible and available participants, rather than seeking statistical representativeness of the entire population.

Moreover, non-probabilistic exploratory sampling is commonly used in research that does not yet have a consolidated mapping of the study universe, or when the investigated phenomenon presents high variability and is in an early stage of understanding [17]. In the case of Agriculture 4.0, technological heterogeneity among farmers, variation in farm size, supply chain, and available infrastructure make it difficult to build a representative probabilistic sample. Furthermore, the emerging nature of the technologies themselves and the lack of comprehensive databases on farmers who use (or are knowledgeable about) these technologies make defining a probabilistic sample unfeasible at an early stage of research. For this reason, the online exploratory approach is considered appropriate, as it allows reaching participants from different regions and production profiles quickly and efficiently.

Although this strategy does not support population inference, it is suitable for the present objective of identifying perceived severity patterns, farm-size differences, and recurring co-occurrence structures among validated barriers. The resulting evidence is therefore analytic and diagnostic rather than representative in a survey-statistical sense.

In this study, 61 responses from farmers were obtained, covering different regions and production profiles in the state of RS. The online questionnaire dissemination was supported by rural unions, cooperatives, and local contact networks, expanding the reach among potential respondents. Data collection was carried out between June and October 2024 through an online questionnaire with voluntary participation. Anonymity, confidentiality, and exclusive scientific use of the data were strictly ensured. The complete questionnaire was included in Appendix A (Table A1).

### 3.5. Statistical Analysis

The analytical strategy combined complementary techniques. Barrier Severity Index (BSI) was used to summarize perceived severity; Cronbach’s alpha assessed internal consistency; Principal Component Analysis (PCA) and K-means clustering explored heterogeneity in response structure; ANOVA tested mean differences across farm-size groups; and proximity-based barrier networks represented patterns of co-occurrence among highly rated barriers. Importantly, the network component is descriptive–it identifies associations in respondent ratings rather than causal interdependence among barriers.

#### 3.5.1. Data Preparation and Cleaning

Upon collection, the dataset was cleaned and standardized to ensure comparability across items and respondents. The main preparation steps are summarized below:

- **Barrier Item Extraction:** All questionnaire items pertaining to perceived “Barriers” (B) are identified by their systematic numerical codes (e.g., “B_1_”, “B_2_”…“B_25_”). This allows for automated extraction and organization of responses corresponding to each distinct barrier.
- **Numeric Encoding and Missing Data Handling:** All barrier ratings are converted to a numeric format. Any non-numeric or ambiguous responses are treated as missing values (NA) and excluded from barrier-specific analyses, thereby ensuring that only valid data contribute to subsequent calculations.
- **Data Filtering (for Stratified Analysis):** Where applicable, the dataset is filtered to produce subgroups (e.g., by farm size or crop type) using relevant demographic columns, supporting segment-specific network or statistical analysis.

#### 3.5.2. Barrier Severity Index (BSI)

To facilitate comparison across individual barriers, a Barrier Severity Index (BSI) was calculated for each item [16]. The BSI rescales the mean Likert response to a 0–1 range, where higher values indicate greater perceived severity and lower values indicate lesser perceived severity. This index is useful for ranking barriers descriptively before examining their relational structure.

(1) $$\mathrm{BS}I_{j}=\frac{1}{n}\sum_{i=1}^{n}\frac{x_{ij}-1}{k-1}$$

where x_ij denotes the Likert response of respondent i to barrier j, m is the maximum value of the Likert scale (here, 5), and n_j is the number of valid responses for barrier j. The BSI is used in this study as a descriptive severity indicator rather than as a latent-variable estimator.

#### 3.5.3. Aggregated Cluster-Wise Analysis

The 25 barriers were also aggregated into five analytical categories—Technological, Economic, Political, Social, and Environmental—to compare broader dimensions of constraint. Cluster-level BSI values were computed as the arithmetic mean of the individual BSIs belonging to each cluster.

(2) $$\mathrm{Cluster}\;{\mathrm{BSI}}_{c}=\frac{1}{m_{c}}\sum_{j\in c}\mathrm{BS}I_{j}$$

This aggregated view does not replace the item-level analysis. Instead, it complements it by revealing whether one domain of barriers tends to be perceived as more severe than others before the more detailed network and farm-size analyses are considered.

#### 3.5.4. Internal Consistency Analysis: Cronbach’s Alpha

To evaluate the internal consistency of the barrier items, Cronbach’s alpha (α) was calculated for the full set of 25 barriers and separately for each thematic barrier group. Cronbach’s alpha can be expressed as α = [k/(k − 1)] [1 − (Σsᵢ^2^/sT^2^)], where k is the number of items, sᵢ^2^ is the variance of item i, and sT^2^ is the variance of the total score. In this study, α is used only as an indicator of internal consistency and not as proof of unidimensionality.

#### 3.5.5. Dimensionality Reduction: Principal Component Analysis (PCA)

Principal Component Analysis (PCA) was employed as an exploratory dimensionality-reduction technique to identify broad axes of variation in respondents’ barrier ratings. In the present study, PCA is used for data summarization and visualization, not for claiming a definitive latent structure of Agriculture 4.0 barriers.

(3) Σv=λv

where Σ is the covariance matrix of the data, v is the eigenvector (principal component) and λ is the eigenvalue (variance explained by the component). The number of principal components retained is determined using the Kaiser criterion (eigenvalues ≥ 1\geq 1 ≥ 1) and visualized via a scree plot. The first two components are typically selected to capture the majority of variance, allowing for effective two-dimensional visualization.

Because the number of respondents is modest relative to the number of items, the PCA results should be interpreted cautiously and within-sample. They are analytically useful for identifying broad perception gradients and supporting the clustering exercise, but they do not establish a universal factorial structure.

#### 3.5.6. Cluster Analysis: K-Means Clustering

K-means clustering was then applied to the PCA-reduced data to summarize heterogeneity in barrier perception profiles. The resulting clusters should be understood heuristically: they provide a compact description of within-sample response patterns rather than definitive farmer typologies.

(4) $$\underset{C}{min}\sum_{i=1}^{k}\sum_{x\in C_{i}}|x-\mu_{i}{|}^{2}$$

where $C_{i}$ is the Cluster i, $\mu_{i}$ is the centroid of cluster i, and x is the data point. This unsupervised learning approach reveals natural groupings within the data, reflecting different “barrier profiles” among the sample. By applying clustering to the PCA-reduced space, the analysis identifies broad within-sample perception profiles and avoids treating respondents as a homogeneous group. Given the modest sample size and the small number of observations in some clusters, the resulting groups are interpreted as suggestive and hypothesis-generating profiles rather than statistically generalizable farmer segments.

#### 3.5.7. Analysis of Variance (ANOVA)

To assess whether perceived barrier severity differed across farm-size groups, one-way ANOVA was performed for each barrier. This test was used to identify barrier items whose mean scores varied significantly among small, medium, and large farms. ANOVA results are interpreted as evidence of group differences in perceived severity, not as evidence of causal mechanisms. In the Results section, test statistics are reported using the standard notation F(df1, df2), followed by the *p*-value.

### 3.6. Barrier Co-Occurrence Network Analysis

A proximity-based barrier co-occurrence network was constructed for each farm-size group. This procedure was inspired by proximity methods used in related network applications [53], but here it is used strictly as a descriptive, variable-centered representation of association patterns among highly rated barriers.

#### 3.6.1. Binary Adoption Matrix

For each farm-size subgroup, the analysis began with a binary matrix M indicating whether respondent i rated barrier j as highly important. This transformation was used to focus the co-occurrence analysis on the upper end of the Likert scale, where respondents explicitly marked a barrier as important or very important.

(5) $$M_{ij}=1\;\mathrm{if}\;\mathrm{Likert}\;\mathrm{score}\geq 4\;\mathrm{otherwise}$$

The binarization rule classifies scores of 4 or 5 as salient barrier perceptions and scores of 1, 2, or 3 as non-salient for the specific purpose of constructing the co-occurrence network. This decision follows the semantic structure of the Likert scale used in the questionnaire: scores 4 and 5 indicate that a respondent explicitly evaluated a barrier as important or very important, whereas score 3 expresses a moderate position and does not provide clear evidence of high salience. Including score 3 as salient would mix moderate perceptions with strong perceived barriers and would increase network density in a way that could obscure the strongest co-reporting patterns. Therefore, the objective of the binary matrix is not to preserve the full ordinal variation in the survey, but to identify which barriers are jointly reported as clearly important by the same respondents. The loss of ordinal information is acknowledged, and the network results are interpreted together with the BSI, PCA, and ANOVA analyses, which retain the original Likert-scale information.

#### 3.6.2. Ubiquity, Co-Occurrence, and Proximity Calculation

Three quantities were then computed: barrier ubiquity, pairwise co-occurrence, and normalized proximity. Ubiquity captures how frequently each barrier is rated as salient within a subgroup; co-occurrence counts how often two barriers are rated as salient by the same respondents; and proximity normalizes co-occurrence by the maximum ubiquity of the pair, thereby giving more meaning to selective association rather than simple prevalence.

- **Ubiquity (**$U_{j}$**)—**This metric quantifies the frequency with which each barrier j is perceived as significant across all respondents:
  (6) $$U_{j}\;=\sum_{i}M_{ij}$$

Higher ubiquity values therefore indicate more widespread perceived salience, whereas higher proximity values indicate that two barriers are more consistently co-reported relative to their marginal frequencies. This distinction is important because a barrier may be common without being especially selective in its associations, and vice versa.

- **Co-occurrence Matrix (**$C_{jk}$**)—**The co-occurrence between barriers j and k is computed as:
  (7) $$C_{jk}\;=\sum_{i}M_{ij}M_{ik}$$

The co-occurrence matrix identifies how many respondents jointly flagged each barrier pair as salient. It is the basic empirical source from which the pairwise proximity relationships are derived.

- **Proximity Matrix (**$\Phi_{jk}$**)—**To normalize co-occurrence by barrier ubiquity, the proximity between barriers is defined as:
  (8) $$\Phi_{jk}\;=\;\frac{C_{jk}}{max\left(U_{j},U_{k}\right)}$$

The resulting symmetric proximity matrix Φ ranges from 0 to 1. In this study, high proximity is interpreted as evidence that the corresponding barriers are frequently rated as important together by the same respondents. It is not interpreted as evidence that one barrier causes, amplifies, or reinforces the other.

#### 3.6.3. Network Thresholding

To improve interpretability, only above-threshold proximity values were retained in the visualized networks. The threshold (Φ > 0.7545) corresponds to the empirical mean of the proximity distribution and was chosen to highlight stronger-than-average associations while filtering weaker links. The threshold is therefore a pragmatic visualization and structure-extraction device, not a significance test or optimal cut-point in a statistical sense.

#### 3.6.4. Network Graph Construction

The filtered matrices were converted into undirected weighted graphs in which nodes represent barriers and edges represent retained proximity relationships. Node color indicates barrier cluster, node size is proportional to ubiquity, and edge width and grayscale intensity reflect proximity strength.

**Nodes**—Each node corresponds to a specific barrier.
**Color**—Nodes are color-coded according to their assigned barrier cluster (Technological, Economic, Political, Social, or Environmental), allowing for immediate visual differentiation and thematic interpretation.
**Size**—The size of each node is set proportional to its ubiquity $U_{j}$, visually emphasizing the most commonly cited barriers within each subgroup. An edge is drawn between two barriers only when their normalized proximity exceeds the threshold. Darker and thicker edges indicate stronger co-reporting among salient barriers. Solid and dashed line types are used only as additional visual cues for stronger versus moderately strong retained ties.
**Color (Grayscale)**—Edge color is mapped to a grayscale gradient, where darker lines represent higher proximity values. This reinforces the interpretation of edge thickness and ensures accessibility in print and black-and-white contexts.
**Line Type**—Edges are rendered as solid lines if $\Phi_{jk}\;>\;0.8$, and as dashed lines otherwise, further distinguishing the strongest empirical associations.
**Node Placement**—To spatially represent the similarity structure implied by the proximity matrix, node coordinates were calculated via classical Multidimensional Scaling (MDS) on the dissimilarity matrix $D_{jk}\;=\;1\;-\;\Phi_{jk}$.

Node positions were initialized using classical Multidimensional Scaling (MDS) on the dissimilarity matrix (1 − Φ) and then refined for readability. Consequently, spatial closeness in the final plots should be interpreted as descriptive similarity in retained proximity patterns, not as a geometric estimate with independent statistical meaning.

#### 3.6.5. Visualization, Legends, and Comparative Analysis

All network plots include cluster and edge-strength legends to support comparison across farm-size groups. Using the same basic rules for all three subgroups makes it possible to compare network density, connectedness, prominent nodes, and association patterns across small, medium, and large farms.

**Node Legends**—Indicate the color mapping for each barrier cluster.
**Edge Legends b**—Depict representative edge widths and gray levels for key proximity values, enabling the viewer to associate visual features with quantitative connection strengths.

**Figure 1:**
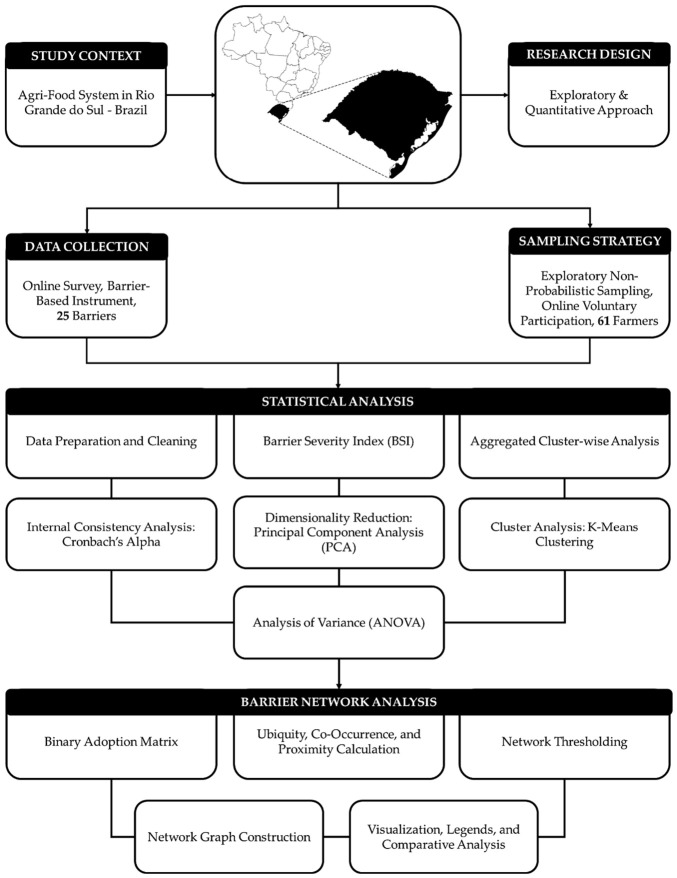
Methodological flow adopted to analyze barriers to Agriculture 4.0 adoption in the agri-food system of Rio Grande do Sul (RS), Brazil, based on an exploratory co-occurrence network analysis.

Figure 1 summarizes the overall methodological workflow, from survey design and data preparation to severity analysis, multivariate exploration, inferential comparison, and descriptive co-occurrence network construction.

## 4. Results

The results combine severity measures, multivariate exploration, inferential comparisons, and farm-size-specific co-occurrence networks. Together, these analyses provide a descriptive account of which barriers are perceived as most severe, which barriers vary by scale of production, and which barriers tend to be jointly rated as important within each subgroup.

### 4.1. Sociodemographic Characteristics

To assess whether perceived barrier severity differed across farm-size groups, one-way ANOVA was performed for each barrier. This test was used to identify barrier items whose mean scores varied significantly among small, medium, and large farms. ANOVA results are interpreted as evidence of group differences in perceived severity, not as evidence of causal mechanisms. In this section, test statistics are reported using the standard notation F(df1, df2), followed by the *p*-value. Educational attainment was comparatively high for an exploratory rural survey, with a substantial share of respondents reporting undergraduate or postgraduate education. Even within this relatively educated sample, however, the later results show that educational gaps and lack of digital skills remain salient barriers—an important point when interpreting the social dimension of Agriculture 4.0 readiness.

The production profile was concentrated in a limited number of systems, with soybean as the most frequent crop, and field-crop production remaining dominant overall. This sample composition should be borne in mind when interpreting the results because the identified barrier configurations are more likely to reflect the realities of the production systems represented most strongly in the sample.

The purpose of the study is not to produce a demographic typology of RS farmers, but to use this sample as the empirical basis for an exploratory analysis of perceived barriers and their co-occurrence structure. Accordingly, the emphasis of the results lies on barrier severity, association patterns, and scale-dependent differences rather than on respondent profiling as an end in itself. The full set of responses and raw data is publicly available for download in PDF format (Link: https://docs.google.com/spreadsheets/d/e/2PACX-1vSum5A-6oBdto_a-dLEahOkgf9kuoHU1Bbo8Qp_GMT5fsmjpRGurxS3eWqj8q1EUEjMKM7IP8fBakE8/pub?gid=1082544757&single=true&output=pdf accessed on 26 January 2026).

### 4.2. Perceived Severity of Barriers to Agriculture 4.0 Adoption

To quantify the relative severity of the various barriers hindering the adoption of Agriculture 4.0 technologies, a Barrier Severity Index (BSI) is computed for each individual barrier. The BSI represents a normalized transformation of the Likert-scale responses provided by participants, where original scores ranged from 1 (strongly disagree) to 5 (strongly agree). This transformation rescales the data to a (0, 1) interval using the following formula:

(9) $$\mathrm{BSI}\;=\;\frac{\bar{X}\;-\;1}{k-1}$$

where $\bar{X}$ is the mean Likert score for the barrier, and k is the number of Likert response levels, which equals 5 in this study. Higher BSI values indicate that participants perceive a given barrier as more severe or problematic in the context of Agriculture 4.0 adoption.

**Figure 2:**
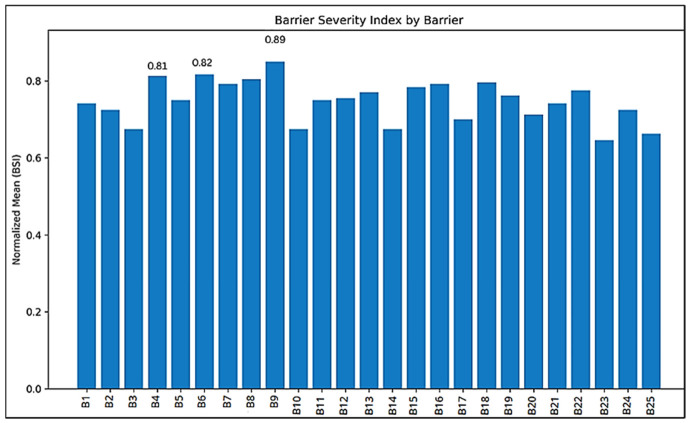
Barrier Severity Index (BSI) for the 25 barriers to Agriculture 4.0 adoption in the agri-food system of Rio Grande do Sul, Brazil.

Figure 2 presents the BSI values for the 25 barriers. The severity pattern indicates that cost- and access-related barriers occupy the highest positions in the ranking, followed by infrastructure and capability-related items. *B_9_—Lack of Affordable Solutions for Farmers* shows the highest BSI (0.89), indicating that cost-effective access to Agriculture 4.0 technologies remains the most severe perceived obstacle in the study sample. *B_6_—High Cost of Facility Maintenance* also appears among the most severe barriers (BSI = 0.82), suggesting that respondents are concerned not only with acquisition costs but also with the ongoing economic burden of implementation, maintenance, and system operation. *B_4_—Lack of Infrastructure* follows closely (BSI = 0.81), reinforcing the importance of rural connectivity and basic digital support conditions in Agriculture 4.0 adoption.

Other high-severity items include *B_8_—High Cost of Operational Components*, *B_7_—High Cost of Skilled Labor*, and *B_18_—Lack of Digital Skills*. Taken together, these scores indicate that Agriculture 4.0 adoption in this sample is constrained not only by the existence of technologies, but also by whether those technologies are affordable, maintainable, supportable, and usable in practice. At the lower end of the ranking, environmental barriers such as *B_23_—Sustainability Constraints* and *B_25_—Limitations in Sustainable Energy Supply* exhibit comparatively lower BSI values. This does not mean that such issues are irrelevant; rather, it suggests that respondents perceived more immediate constraints in cost, infrastructure, implementation, and skills.

### 4.3. Cluster-Wise Barrier Severity Index (BSI)

**Figure 3:**
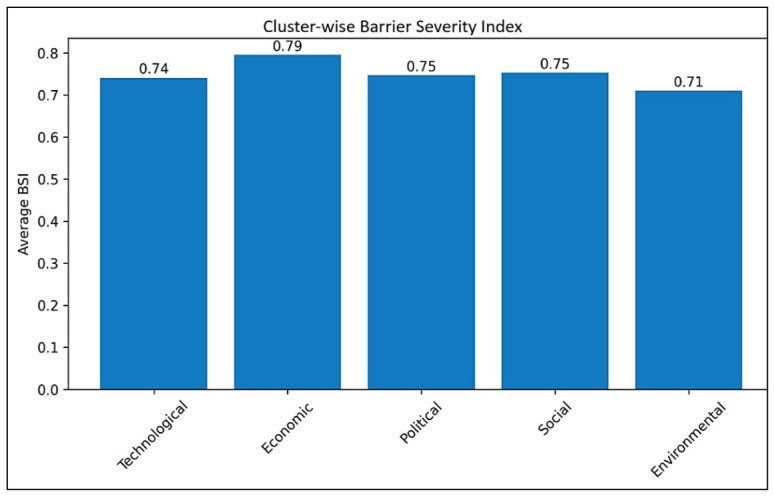
Average Barrier Severity Index (BSI) by thematic cluster in the agri-food system of Rio Grande do Sul, Brazil.

To complement the item-level BSI ranking, the barriers were aggregated into five thematic categories. Figure 3 shows that the severity pattern remains differentiated at the cluster level as well.

The Economic cluster records the highest average BSI (0.79), confirming that cost structures and financial feasibility are the dominant perceived constraints in the sample.

This result is substantively important because it indicates that the economic problem is not limited to a single item such as acquisition cost. Instead, it spans affordability, component cost, maintenance burden, and labor cost, thereby constituting a broader domain of adoption difficulty.

The Social and Political clusters follow closely (both around 0.75). Their proximity to the Economic cluster suggests that perceived Agriculture 4.0 constraints are not exclusively financial; they also involve capabilities, information, organizational support, and implementation conditions.

The Technological cluster is only slightly lower (0.74), which indicates that technical challenges remain relevant but are not perceived in isolation from broader economic and institutional conditions.

The Environmental cluster has the lowest average BSI (0.71). This finding should be interpreted cautiously. Lower average severity does not imply absence; rather, it suggests that respondents prioritize more immediate operational and economic constraints when evaluating adoption barriers.

Taken together, the cluster-level results do not justify a causal claim that one domain reinforces another. They do, however, show a clear ordering of perceived severity and motivate closer examination of how highly rated barriers are associated in the network analysis.

Overall, the BSI results establish a consistent descriptive baseline for the remainder of the paper–economic barriers are the most severe on average, but social, political, and technological domains remain close enough in magnitude to suggest that adoption is experienced as a multidimensional problem rather than a single-cost problem.

This multidimensionality becomes especially important when the analysis moves from severity to co-occurrence. A barrier may have a high BSI because it is broadly salient, but its role in the network depends on which other barriers tend to be reported alongside it. For this reason, the cluster averages are best interpreted as an entry point into the barrier landscape, whereas the farm-size-stratified networks reveal how that landscape is structured in respondents’ perceptions. In other words, BSI identifies what is severe; the co-occurrence networks help show what tends to be perceived together.

### 4.4. Reliability Analysis (Cronbach’s Alpha)

Cronbach’s alpha (α) was computed for each of the five predefined barrier categories—Technological, Economic, Political, Social, and Environmental—as well as globally, taking into account all 25 barrier items together, in order to evaluate the internal reliability of the barrier measurement tool. A commonly used indicator of internal consistency, Cronbach’s alpha shows how well individual items in a group correlate with one another and helps quantify a shared latent concept.

#### 4.4.1. Global Reliability

According to standard psychometric standards, the total Cronbach’s alpha for the entire set of 25 barriers was calculated to be 0.903, indicating strong internal consistency [56,57]. This high result indicates that the barrier items work well together as a trustworthy scale for identifying the perceived barriers to the implementation of Agriculture 4.0. The scale shows enough coherence to effectively summarize respondents’ attitudes and views, which supports the methodological approach of combining discrete barrier items into larger constructs.

#### 4.4.2. Cluster-Wise Reliability

Cronbach’s alpha is calculated independently for each of the five Agriculture 4.0 barrier categories in order to obtain a more detailed understanding of scale reliability within particular dimensions of the barriers. The resulting reliability coefficients for each cluster are presented in Table 2.

The reliability analysis indicates that the proposed cluster structure provides a consistent measurement framework for analyzing perceived constraints to Agriculture 4.0 adoption in the agri-food system. As shown in Table 2, most clusters present acceptable to strong internal consistency, supporting their use as aggregated analytical dimensions.

The Technological (α = 0.828), Social (α = 0.829), and Political (α = 0.783) clusters exhibit solid reliability levels, suggesting that respondents assess these dimensions in a coherent and structured manner. The Economic cluster also reaches acceptable reliability (α = 0.744), which is consistent with the exploratory nature of the study and indicates stable perceptions regarding cost- and investment-related constraints.

The Environmental cluster shows the lowest internal consistency among the five dimensions (α = 0.673). This comparatively lower value likely reflects greater variability and heterogeneity among the environmental items included in the cluster. Because the value remains within an acceptable range for exploratory research, the cluster was retained, but its interpretation should be more cautious than that of the clusters with stronger internal consistency.

#### 4.4.3. Implications

The reliability estimates indicate that the barrier measurement instrument is psychometrically adequate for the study context. The overall Cronbach’s alpha reflects a robust scale for assessing adoption challenges related to Agriculture 4.0 technologies. At the construct level, the Technological, Economic, Political, and Social clusters exhibit acceptable to strong internal consistency, supporting their use in subsequent analyses such as Principal Component Analysis (PCA), clustering, and Barrier Severity Index (BSI) calculations. Although the Environmental dimension presents comparatively lower reliability, its alpha remains within an acceptable range for exploratory research. Overall, the results confirm that the instrument provides a consistent empirical basis for analyzing perceived barriers to the adoption of Agriculture 4.0.

### 4.5. Principal Component Analysis (PCA)

Principal Component Analysis (PCA) was applied to explore the latent structure underlying the perceived barriers to the adoption of Agriculture 4.0 technologies. As a dimensionality reduction technique, PCA transforms the original set of correlated barrier variables into a smaller number of uncorrelated components, while preserving most of the variance contained in the data. This approach allows for a more parsimonious representation of complex multivariate relationships and supports subsequent exploratory analyses.

**Figure 4:**
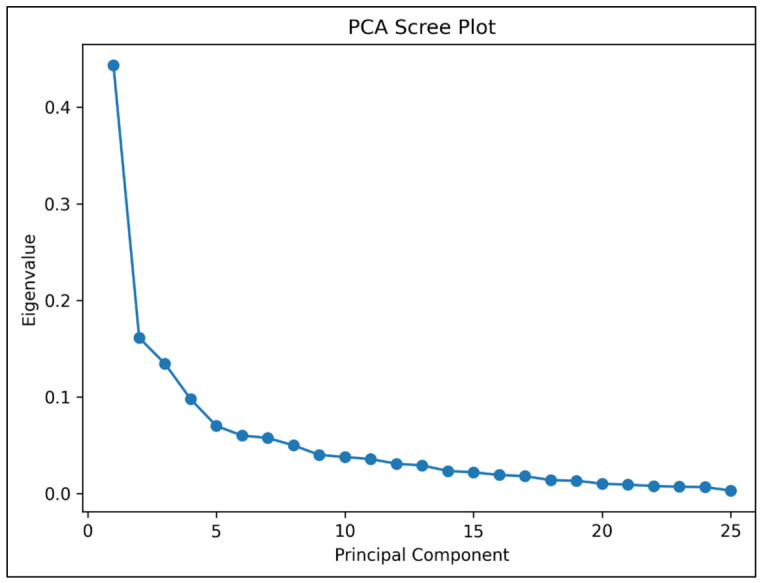
Scree plot of principal components for Agriculture 4.0 adoption barriers.

Figure 4 presents the scree plot of the eigenvalues associated with the extracted principal components. The graphical distribution shows a clear inflection point after the second component, followed by a marked decline in the magnitude of the eigenvalues. This pattern indicates that the first two components account for the most substantial share of variance in the data, while the remaining components contribute only marginally to explaining the underlying structure. Based on this criterion, two principal components were retained for interpretation and subsequent analyses.

The first Principal Component (PC1) accounts for approximately 44.6% of the total variance and represents the dominant underlying dimension structuring respondents’ perceptions of adoption barriers. This component reflects a generalized barrier intensity, capturing the common variance shared across multiple constraint categories.

The second Principal Component (PC2) explains an additional 16.3% of the variance and captures secondary but still meaningful contrasts in the data, reflecting differentiated patterns in how specific barriers are perceived relative to the dominant dimension represented by PC1. From the third component onward, each principal component individually explains less than 10% of the variance, indicating a diminishing marginal contribution to the overall explanatory power.

Taken together, the first two principal components account for approximately 60.9% of the total variance. This cumulative share suggests that the multidimensional structure of perceived barriers to Agriculture 4.0 adoption can be adequately represented in a two-dimensional component space. In line with established PCA interpretation criteria—namely the cumulative variance threshold and the identification of the elbow point in the scree plot—the PC1–PC2 solution was therefore retained for subsequent cluster analysis and graphical representations.

#### 4.5.1. PCA-Reduced Data and K-Means Clustering

The scores associated with the first two principal components (PC1 and PC2) were subsequently used as input for a K-Means clustering procedure in order to identify distinct patterns in respondents’ perceptions of barriers to Agriculture 4.0 adoption. By operating on PCA-reduced data, the clustering process focuses on the most informative dimensions of variance, preserving the dominant structural patterns in the dataset while reducing noise and redundancy among the original variables.

Based on exploratory diagnostics—including visual inspection of the distribution of observations in the PC1–PC2 space and the interpretability of the resulting group profiles—the number of clusters was set to k = 3. This configuration provided a parsimonious yet meaningful representation of heterogeneity in barrier perceptions, yielding clearly distinguishable and analytically interpretable clusters.

#### 4.5.2. Distribution and Interpretation of Clusters

**Figure 5:**
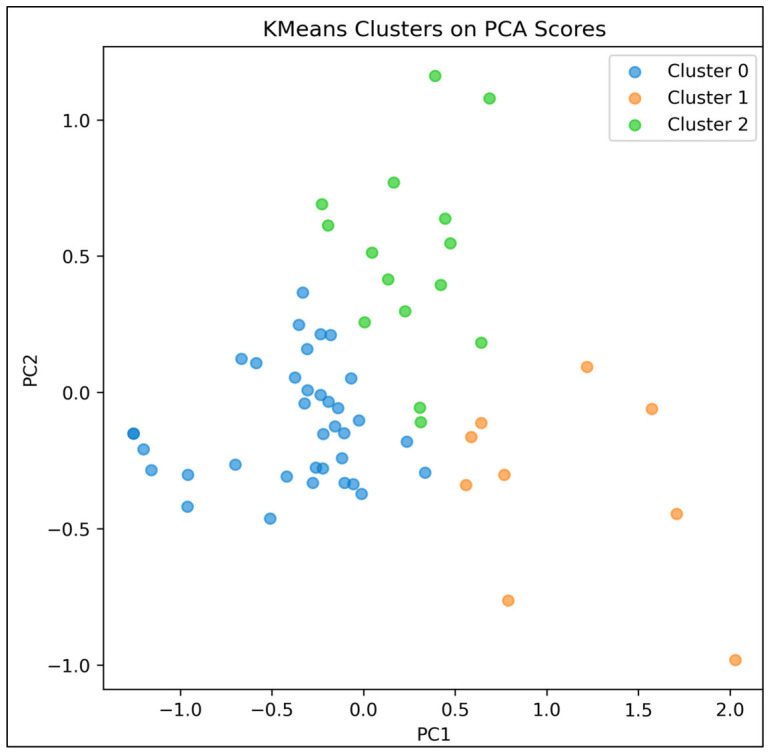
Distribution of respondent clusters in the PC1–PC2 space derived from PCA.

Table 3 and Figure 5 summarize the three-cluster solution derived from PCA-reduced data. The clustering result is useful for describing within-sample heterogeneity, but it should be interpreted as exploratory rather than as a definitive segmentation of Brazilian farmers.

Figure 5 depicts the spatial configuration of the three clusters in the two-dimensional PC1–PC2 space derived from PCA, visually reinforcing the distinct perception profiles identified through K-Means clustering. The value of the analysis with even a small number of respondents lies in showing that respondents occupy different regions of the PCA perception space, thereby complementing the item-level BSI, ANOVA, and co-occurrence network analyses.

Cluster 1 comprises respondents with lower scores on the dominant PCA dimension, indicating a stronger concentration of perceived core adoption constraints.

Cluster 2 is more differentiated along the second component, suggesting greater sensitivity to a secondary bundle of barriers related to learning, institutional support, and capability conditions.

Cluster 0 lies closer to the center of the PCA space and represents respondents with more moderate and relatively balanced barrier evaluations.

These clusters are analytically helpful because they show that the barrier landscape is not completely homogeneous across respondents. At the same time, their meaning should not be overstated: they summarize within-sample response structure and do not by themselves establish stable farmer typologies beyond the present dataset.

The PCA and clustering results therefore serve as supportive exploratory evidence of heterogeneity, complementing—but not replacing—the more concrete item-level and network-based analyses.

### 4.6. Statistical Testing (ANOVA)

A series of one-way ANOVA tests was conducted to examine whether mean perceived severity differed across farm-size groups. These results identify which specific barriers vary significantly by scale and therefore complement the descriptive co-occurrence networks, which focus on association structure within each subgroup. Also, the results are reported using the standard F(df1, df2) format, where df1 represents the between-group degrees of freedom and df2 represents the residual degrees of freedom. For tests using all 61 valid responses, df1 = 2 and df2 = 58.

#### 4.6.1. B_1_—Technological Complexity

*Definition:* Barrier B1 refers to difficulties arising from the low usability of Agriculture 4.0 technologies within the agri-food system, including autonomous machines, sensors, applications, and software for agricultural data collection and analysis. These challenges are commonly associated with insufficient education and training, inadequate ergonomics, or poorly designed user interfaces.

F(2, 58) = 5.46; *p*-value = 0.0068

The significant result indicates that perceived technological complexity is not uniform across farm sizes. This suggests that usability, interface demands, and operational difficulty are experienced differently depending on the production scale and organizational capacity of the farm.

#### 4.6.2. B_2_—Incompatibility Between Components

*Definition:* Barrier B_2_ captures constraints related to the integration of equipment and software from different technology providers with existing agricultural operations, including limited interoperability among heterogeneous sensors and digital platforms in the agri-food system.

F(2, 58) = 5.02; *p*-value = 0.0098

The significant difference for B_2_ shows that interoperability and component integration are also scale-sensitive. Farms do not face digital integration challenges in the same way, and this becomes especially relevant in the later network analysis of medium and large farms.

#### 4.6.3. B_4_—Lack of Infrastructure

*Definition:* Barrier B_4_ reflects insufficient telecommunications and digital infrastructure on farms, highlighting the need for robust connectivity in rural areas to enable the effective implementation of Agriculture 4.0 technologies.

F(2, 58) = 3.25; *p*-value = 0.0460

The result for B_4_ confirms that digital and telecommunications infrastructure remains unequally experienced across farm sizes. Lack of connectivity and infrastructural support is therefore not a background condition alone; it is an explicitly differentiated adoption barrier.

#### 4.6.4. B_6_—High Cost of Facility Maintenance

*Definition:* Barrier B_6_ refers to the high investment and operational costs required to establish and maintain Agriculture 4.0 systems in the agri-food system, including machinery, software solutions, and telecommunications networks.

F(2, 58) = 5.27; *p*-value = 0.0080

The ANOVA result for B_6_ reinforces the central role of economic feasibility. Maintenance and operating costs are perceived differently across scales, indicating that the economics of sustained Agriculture 4.0 use vary substantially with production structure.

#### 4.6.5. B_16_—Educational Gaps

*Definition:* Barrier B_16_ highlights inadequacies in agricultural education systems regarding the competencies required for Agriculture 4.0, including digital skills, data analysis capabilities, and technology management.

F(2, 58) = 3.35; *p*-value = 0.0421

The significant variation in B_16_ suggests that educational and training-related deficits are also scale-dependent. This finding later helps explain why capability-related barriers become more visible in some of the subgroup networks.

#### 4.6.6. B_24_—Limited Farm-Level Data Collection Techniques

Definition: Barrier B_24_ reflects challenges in developing and implementing efficient and reliable techniques for collecting high-quality data directly from farms within the agri-food system.

F(2, 58) = 8.45; *p*-value = 0.0006

B_24_ shows the strongest farm-size difference among the tested items. This is an important result because reliable on-farm data collection is a functional precondition for many Agriculture 4.0 technologies; unequal perceptions of this barrier point directly to a scale-related digital divide.

Taken together, the ANOVA results show that farm size matters not only for cost and infrastructure, but also for interoperability, capability formation, and practical data generation.

Conversely, the absence of statistically significant differences for some other barriers indicates that not all constraints are scale-specific. Some perceived difficulties appear relatively widespread across the sample regardless of farm size.

The ANOVA results therefore provide a useful bridge to the network analysis: they show where mean severity differs across groups, while the networks show how highly rated barriers are associated within each group.

### 4.7. Network Structure of Agriculture 4.0 Barriers by Farm Size

To visualize how highly rated barriers are associated within each farm-size group, proximity-based co-occurrence networks were constructed for small (≤20 ha), medium (21–100 ha), and large (>100 ha) farms. The networks should be read descriptively: edges indicate frequent co-reporting among salient barriers, not causal influence, mutual reinforcement, or actual interaction between barriers in the field.

- Node color identifies barrier cluster (Technological, Economic, Political, Social, or Environmental).
- Node size is proportional to ubiquity, that is, the frequency with which the barrier was rated as important or very important within the subgroup.
- Edge width reflects retained proximity strength.
- Darker edges represent stronger retained co-occurrence among salient barriers.
- Solid lines indicate especially strong retained ties, whereas dashed lines indicate moderately strong retained ties above the empirical threshold.

Node placement is based on proximity-derived similarity and should therefore be interpreted as a visual aid to association structure rather than as a standalone statistical result.

#### 4.7.1. Barrier Network for Small Farms (≤20 ha)

**Figure 6:**
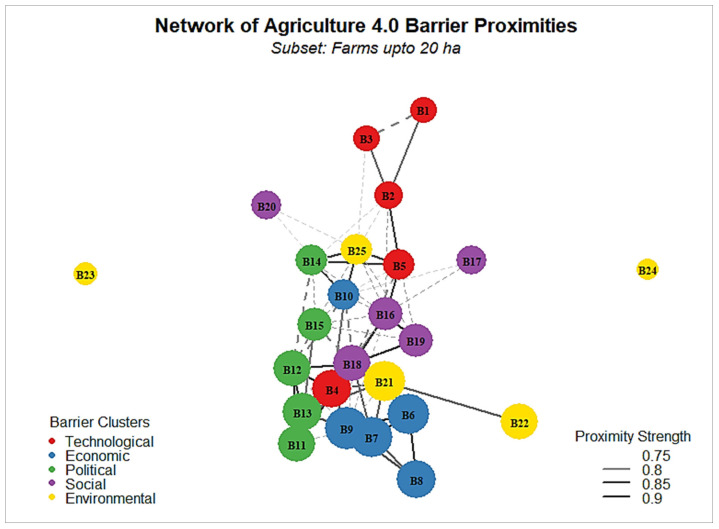
Barrier network for small farms (≤20 ha).

The small-farm network (Figure 6) is the sparsest of the three subgroup networks. After thresholding, it contains 89 edges, a density of 0.2967, and three connected components, with B23 and B24 isolated from the main structure. This already suggests that perceived barriers among small farms are not organized into a single fully integrated configuration.

In terms of ubiquity, *B_9_—Lack of Affordable Solutions for Farmers*—is the most widespread small-farm barrier, followed by *B_6_—High Cost of Facility Maintenance*, *B_7_—High Cost of Skilled Labor*, and *B_21_—Climate and System Behavior Challenges*. The strongest retained ties include B_6_–B_8_, B_11_–B_12_, B_16_–B_19_, B_6_–B_9_, and B_6_–B_7_. Substantively, these patterns indicate two linked but still distinguishable structures: an economic-technological affordability block and an institutional-capability-information block.

This is an important correction to a purely cost-centered reading. Small farms certainly face affordability and infrastructure problems, but the network also shows strong associations among B_11_, B_12_, B_13_, B_16_, B_18_, and B_19_. In other words, limited availability, lack of farmer-centered support, weak implementation planning, educational gaps, digital skills deficits, and information asymmetry are not peripheral add-ons; they form a visible associated cluster in respondents’ perceptions.

Rather than supporting a claim that barriers causally reinforce one another, the small-farm network suggests that respondents often perceive affordability, operational feasibility, institutional support, and digital capability as part of the same adoption difficulty. This segmented pattern is consistent with a context in which basic access and implementation support remain tightly entangled.

#### 4.7.2. Barrier Network for Medium Farms (21–100 ha)

**Figure 7:**
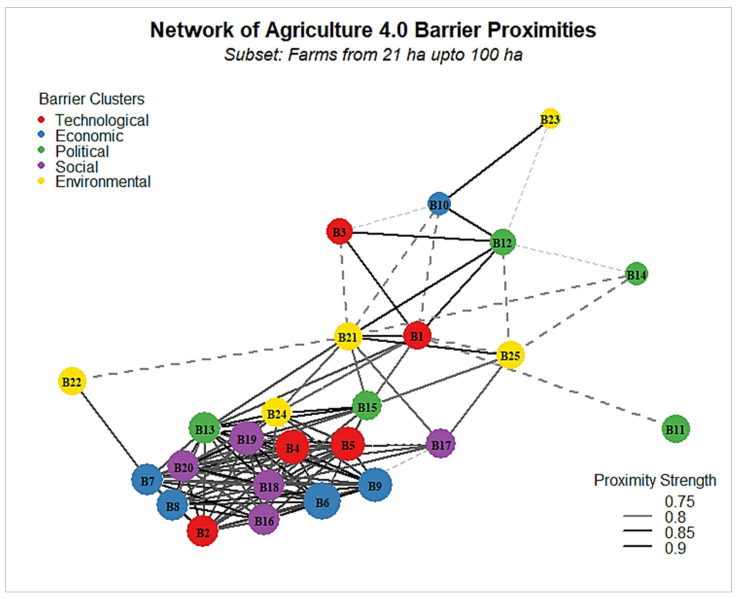
Barrier network for medium farms (21–100 ha).

The medium-farm network (Figure 7) is more integrated than the small-farm network. It contains 132 edges, a density of 0.44, and forms a single connected component. This means that all 25 barriers remain linked, directly or indirectly, within one co-occurrence structure after thresholding. The most ubiquitous medium-farm barriers include B_4_, B_6,_ B_5_, B_9_, and B_19_. The strongest retained ties include B_4_–B_6_, B_5_–B_6_, B_6_–B_9_, B_6_–B_19_, B_4_–B_19,_ B_4_–B_5_, B_4_–B_7_, B_4_–B_9_, B_5_–B_13_, and B_9_–B_16_. In addition, node-level metrics place B_19_, B_5_, B_4_, B_13_, B_9_, and B_6_ among the most connected or strongest positions in the network.

Taken together, these results suggest that medium farms exhibit the most globally integrated barrier configuration in the sample. Cost, infrastructure, reliability, information asymmetry, implementation planning, and digital capability appear jointly embedded within one association structure rather than split into separate subnetworks.

This pattern is consistent with interpreting medium farms as a transitional adoption segment. These farms are not limited only by basic access constraints, but neither have they fully decoupled technical, organizational, and capability-related difficulties. Instead, they tend to report a broad bundle linking affordability, trust in digital systems, infrastructure, information, and implementation readiness.

It is therefore more accurate to say that medium farms display an integrated co-occurrence structure than to say that barriers mutually reinforce one another. The evidence shows patterned association in perception, not directional causality.

Political and social barriers are especially notable in this subgroup because they are not outside the main structure; they are woven into it. Items such as *B_13_—Lack of Clear Action Plans for Technology Implementation* and *B_18_—Lack of Digital Skills* occupy visibly connected positions, which suggests that organizational preparedness and capability formation are central parts of the adoption problem at this scale.

Environmental barriers are less dominant than economic and technological ones, but they are not absent. Their participation in the connected network indicates that environmental operating issues remain part of the broader perceived constraint structure, even if they are not the main organizing theme.

#### 4.7.3. Barrier Network for Large Farms (>100 ha)

**Figure 8:**
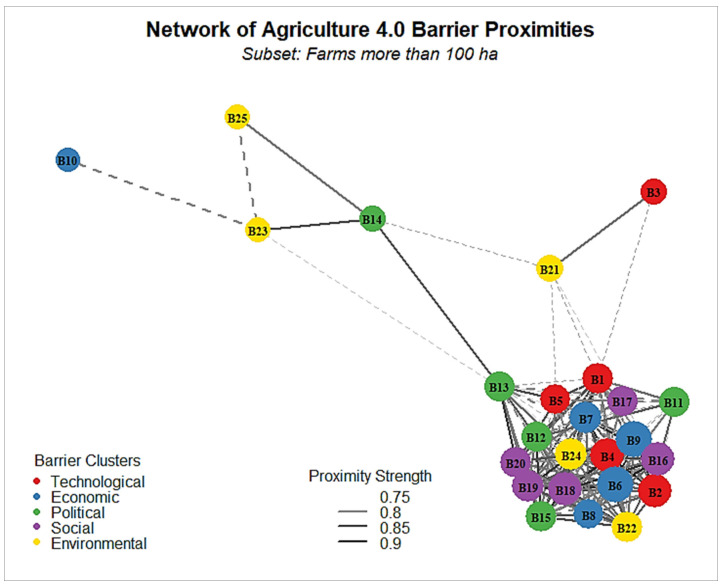
Barrier network for large farms (>100 ha).

The large-farm network (Figure 8) is the densest of the three in retained edge count and density, with 151 edges and a density of 0.5033. At the same time, it is not fully connected: B_10_ and B_25_ are isolated after thresholding. This combination—high density within the main structure plus isolated peripheral items—suggests a dense but differentiated configuration rather than a uniform one.

Barrier ubiquity is also high in this subgroup. B_9_ is universal in the thresholded large-farm matrix, while B_6_, B_2_, B_16_, and B_18_ are also highly prevalent. Among the strongest retained ties are B_6_–B_9_, B_12_–B_15_, B_13_–B_14_, B_2_–B_9_, B_9_–B_16_, B_9_–B_18_, B_2_–B_6_, B_4_–B_6_, and B_16_–B_18_. Node metrics further highlight the strong positions of B_16_, B_20_, B_7_, B_12_, B_18_, B_9_, B_2_, B_1_, B_22_, and B_15_.

These results show that large farms are not simply constrained by cost in a narrow sense. Instead, they report a dense association structure in which affordability and maintenance costs are closely tied to interoperability, digital skills, educational gaps, organizational adaptation, and governance-related items.

The large-farm configuration is therefore best interpreted as a mature but still demanding adoption environment. Once some basic access constraints are partly overcome, the barrier landscape does not disappear; rather, it becomes more differentiated around integration, coordination, skills, governance, and system management.

Political and social barriers are especially important here because they sit close to the economic-technological core rather than far from it. Strong ties such as B_12_–B_15_ and B_13_–B_14_ indicate that farmer-centered support structures, R&D and business-model development, action planning, and regulatory/data-governance issues are perceived together in the large-farm setting.

Environmental barriers are heterogeneous in this subgroup. B_22_ remains well integrated into the main structure, whereas B_25_ is isolated after thresholding. This suggests that environmental and data-quality issues should not be described uniformly as peripheral; some are embedded in the core co-occurrence structure, whereas others are not.

Overall, the large-farm network supports a differentiated reading of scale: greater production scale does not eliminate barriers, but it changes the kinds of associations that dominate the perceived adoption landscape.

#### 4.7.4. Cross-Scale Comparison of Barrier Co-Occurrence Networks

Comparing the three networks reveals both continuity and structural variation across scales. First, economic barriers remain prominent in all groups. B_9_ and B_6_ repeatedly appear either among the most ubiquitous barriers or within the strongest retained ties, which confirms that affordability and maintenance are stable core concerns across the sample.

Second, the networks differ in overall structure. Small farms display the sparsest and most segmented pattern, medium farms the most globally integrated connected structure, and large farms the densest but more differentiated structure with isolated peripheral items.

Third, the role of social and political barriers changes with scale. In small farms, these barriers form a visible cluster alongside affordability constraints; in medium farms, they are integrated into the main connected structure; and in large farms, they become closely tied to the differentiated core of interoperability, skills, and governance.

Fourth, environmental barriers are not uniformly peripheral. Their role varies by subgroup and by item. Some environmental barriers remain marginal or isolated after thresholding, whereas others—especially those linked to data effectiveness or operating conditions—are associated with the broader adoption structure.

These comparisons strengthen the descriptive contribution of the network analysis. They show that the perceived barrier landscape is not identical across scales of production, even when some high-severity items recur across all groups.

In short, the co-occurrence evidence suggests that different farm sizes face different configurations of associated constraints. This insight is more analytically precise than a general statement that all barriers form one interdependent system.

## 5. Discussion

The results of this study support an overarching conclusion–barriers to Agriculture 4.0 adoption in Rio Grande do Sul are multidimensional and patterned, but they should not be interpreted as a causally established system of mutual reinforcement on the basis of the present design alone. What the study shows is that farmers’ perceptions are structured in recurring ways. Some barriers are rated as more severe than others, some differ significantly by farm size, and some tend to be reported together with notable regularity within farm-size groups.

At the severity level, economic barriers dominate. *B_9_—Lack of Affordable Solutions for Farmers* and *B_6_—High Cost of Facility Maintenance*—occupy especially prominent positions in the BSI ranking, while *B_4_—Lack of Infrastructure*—confirms that the economic problem is tightly linked to enabling conditions rather than to prices alone. This finding is consistent with a large body of literature showing that Agriculture 4.0 adoption depends not only on technological availability but on financially viable implementation conditions [14,15,16,17,18,58,59,60,61,62,63,64].

The BSI results also indicate, however, that Agriculture 4.0 adoption cannot be reduced to a simple cost narrative. Social and political clusters remain close to the economic cluster in average severity, and technological barriers remain only slightly lower. This means that affordability, capability formation, information, governance, and technical feasibility all matter within the same empirical landscape.

The ANOVA results deepen this interpretation by showing that several key barriers vary significantly by farm size. In particular, B_1_, B_2_, B_4_, B_6_, B_16_, and B_24_ differ across small, medium, and large farms. These results confirm that production scale is not merely a background characteristic; it shapes how farmers perceive complexity, interoperability, infrastructure, maintenance costs, education, and on-farm data collection.

The network results add a further layer by showing that highly rated barriers are associated differently across farm-size groups. This is the main analytical contribution of the study. Instead of assuming that the same barrier landscape applies equally to all farms, the co-occurrence networks reveal different subgroup-specific structures.

For small farms, the network suggests a segmented configuration in which affordability and technical access are closely linked to institutional support, planning, and digital capability. This is consistent with a setting in which farmers may perceive Agriculture 4.0 not simply as expensive, but as difficult to access, difficult to operationalize, and weakly supported by the surrounding institutional environment.

For medium farms, the evidence points to the most integrated overall association structure. Cost, infrastructure, reliability, information asymmetry, planning, and skills are all tied into one connected network. This indicates that medium farms may face a particularly bundled transition problem: they are far enough along to confront implementation and organizational issues, but not far enough along to separate those issues from basic cost and infrastructure concerns.

### 5.1. Farm Size and Structural Heterogeneity in Adoption

Taken together, the severity, ANOVA, and network results show that farm size is a major axis of heterogeneity in perceived Agriculture 4.0 barriers. Small farms face a more segmented but fragile structure, medium farms face the most integrated structure, and large farms face a dense but more differentiated one.

This finding is important because it challenges the assumption that larger scale simply means fewer barriers. The large-farm network demonstrates that many constraints persist even when farms are more capitalized. What changes is not the existence of barriers, but the configuration in which they are perceived—moving from basic access concerns toward integration, interoperability, coordination, skills, and governance.

The especially strong farm-size variation observed for *B_24_—Limited Farm-Level Data Collection Techniques*—further reinforces the existence of a scale-dependent digital divide. Reliable farm-level data are foundational for many Agriculture 4.0 applications, so unequal perceptions of this barrier point directly to unequal technological readiness across farm structures.

### 5.2. Interpreting Barrier Co-Occurrence Structures

A central implication of the study is conceptual. The barrier networks should be understood as co-occurrence structures in respondent perception, not as direct evidence that barriers causally intensify one another. The present design is cross-sectional, survey-based, and correlational in its network component. It shows which barriers are frequently rated together, not whether one barrier produces another.

This distinction does not weaken the contribution of the study; rather, it sharpens it. Descriptive co-occurrence mapping is valuable because it identifies which constraints are likely to be encountered together from the farmers’ point of view. That insight is highly relevant for intervention design even when causal ordering remains unknown.

The results therefore support statements about associated barrier bundles, recurring co-reporting patterns, and farm-size-dependent configurations of perceived constraints. They do not support strong claims about cumulative mechanisms, systemic reinforcement, or directional interdependence without additional longitudinal, experimental, or process-tracing evidence.

### 5.3. Implications for Public Policies and Management Strategies

A practical implication of this study is that interventions aimed at promoting Agriculture 4.0 adoption should not be designed as single-item solutions. A subsidy for equipment, for example, may have limited effect if infrastructure, implementation support, trusted information, and digital skills remain unresolved. The network results suggest that barrier bundles vary by scale and should therefore be addressed with scale-sensitive policy mixes.

For small farms, the most appropriate interventions are likely to combine affordability measures with basic enabling conditions. These may include subsidized or shared access models, cooperative purchasing, digital-extension support, connectivity expansion, and farmer-centered institutional arrangements that reduce informational and organizational uncertainty alongside direct cost burdens.

For medium farms, the results point to a need for coordinated transition support. Financial instruments remain important, but so do implementation roadmaps, trusted advisory services, training, and mechanisms that reduce the burden of maintaining and integrating digital systems. Medium farms appear especially sensitive to barrier bundles that combine cost with readiness and capability.

For large farms, the evidence points toward more advanced intervention needs. Affordability and maintenance do not disappear, but they are increasingly associated with interoperability, governance, action planning, organizational adaptation, and advanced capability formation. Policy and management responses for this group should therefore emphasize standards, data governance, integration support, and innovation-oriented institutional ecosystems.

Across all farm sizes, the stable prominence of economic barriers suggests that affordability remains the common denominator of the adoption problem. However, the network evidence makes clear that affordability is embedded in wider configurations and should not be treated as a standalone policy target.

Overall, these findings indicate that effective policy design should move beyond uniform incentives and instead adopt differentiated, system-aware strategies that reflect the distinct combinations of constraints faced by different farm scales. Such an approach increases the likelihood that support measures will address not only individual barriers, but also the interconnected sets of limitations that shape Agriculture 4.0 adoption decisions.

### 5.4. Conceptual and Methodological Contributions

Conceptually, the study contributes by reframing the analysis of Agriculture 4.0 barriers away from purely isolated item rankings and toward an explicitly relational, but still cautious, interpretation of perception data. The contribution is not a demonstration of social network dynamics among actors; it is a network-analytic representation of how validated barriers are associated in farmers’ responses.

Methodologically, the study combines severity analysis, internal-consistency testing, multivariate exploration, inferential comparison, and descriptive co-occurrence mapping in one integrated framework. This combination makes it possible to examine barriers at several levels at once: average severity, broad cluster patterns, latent response gradients, subgroup differences, and association structure.

The framework is especially useful for exploratory research because it preserves the strengths of conventional survey analysis while adding a structural representation of barrier configurations. It can be adapted to other regions, other agri-food systems, and future longitudinal designs that test whether the observed co-occurrence patterns remain stable over time.

### 5.5. Limitations and Future Research

This study has several limitations. First, it is based on an exploratory regional sample and does not support broad population generalization. The sample is also concentrated in a limited set of production systems, which means that the reported barrier configurations are more likely to reflect the realities of the farming systems most represented in the dataset than those of all Brazilian agricultural sectors.

Second, the network analysis is based on dichotomized Likert responses and an empirical proximity threshold selected to highlight stronger-than-average co-occurrence patterns. This choice has a clear descriptive rationale—isolating highly salient barriers and improving visual interpretability—but it also discards part of the original ordinal information. Future studies should examine robustness using alternative cutoffs, weighted formulations based on the full Likert scale, and sensitivity analyses across threshold choices.

Third, the study is cross-sectional. It therefore cannot determine whether the observed co-occurrence patterns remain stable over time, whether some barriers precede others in real adoption trajectories, or whether specific interventions change the structure of perceived constraints.

Finally, it is important to emphasize that the methodological contribution should be interpreted with caution. The proximity networks do not address issues of causality, threshold sensitivity, or information loss resulting from dichotomization. Their primary value lies in descriptive structure identification and in generating hypotheses for future research.

## 6. Conclusions

This study examined Agriculture 4.0 adoption barriers in the agri-food system of Rio Grande do Sul through an exploratory barrier co-occurrence perspective. Using survey data, BSI, reliability testing, PCA, K-means clustering, ANOVA, and farm-size-stratified proximity networks, the analysis identified both the severity of individual barriers and the distinct association structures through which highly rated barriers are jointly perceived. The results show that economic barriers remain the most severe overall, especially lack of affordable solutions, maintenance costs, and infrastructure deficits. ANOVA results further demonstrated that farm size significantly affects perceptions of technological complexity, interoperability, infrastructure, maintenance costs, educational gaps, and farm-level data collection limitations, reinforcing the existence of scale-dependent heterogeneity in Agriculture 4.0 adoption.

The network analysis showed that barriers are not perceived uniformly across farm sizes. Small farms exhibited the sparsest and most segmented structure, linking affordability constraints to institutional support, planning, and digital capability barriers. Medium farms displayed the most integrated network structure, combining cost, infrastructure, implementation readiness, information asymmetry, and skills within a single connected configuration. Large farms exhibited the densest but more differentiated structure, in which affordability and maintenance costs were closely associated with interoperability, governance, organizational adaptation, and advanced digital skills. These findings should be interpreted descriptively rather than causally. The study does not demonstrate that barriers directly reinforce one another; instead, it shows that farmers in different farm-size groups tend to perceive specific barriers together with notable regularity.

The findings have practical and methodological implications. From a policy perspective, the results suggest that Agriculture 4.0 adoption is unlikely to be accelerated through isolated interventions focused solely on equipment costs. Effective strategies should instead address scale-specific configurations of technological, economic, political, social, and environmental barriers. Methodologically, the study demonstrates how proximity-based co-occurrence networks can complement conventional survey approaches by revealing structural patterns in how barriers are jointly perceived. Future research should test the stability of these configurations using larger and more representative samples, longitudinal designs, and alternative thresholding and weighting strategies. Overall, the evidence suggests that Agriculture 4.0 barriers in this regional Brazilian context are better understood as recurring and farm-size-dependent configurations of associated constraints rather than as isolated and independent adoption barriers.

## Figures and Tables

**Table 1:** Barriers to the adoption of emerging Agriculture 4.0 technologies in the agri-food system.

ID	Barrier	Description	Cluster
B_1_	Technological Complexity	Difficulties arising from the low usability of technological equipment by actors in the agri-food system (e.g., autonomous machines, sensors, apps, and software for agricultural data collection and analysis). These challenges may result from insufficient education and training, as well as inadequate ergonomics or poorly designed user interfaces.	Technological
B_2_	Incompatibility between Components	Constraints related to the integration of equipment and software from different technology providers with existing agricultural operations, including limited interoperability among heterogeneous sensors and digital platforms within the agri-food system.	
B_3_	Energy Management Problems	Limitations associated with energy availability and power consumption that hinder the adoption of Agriculture 4.0 technologies in the agri-food system, particularly regarding battery life and operational autonomy of drones and autonomous robots.	
B_4_	Lack of Infrastructure	Insufficient telecommunications and digital infrastructure on farms, highlighting the need for robust digital connectivity in rural areas to support the agri-food system.	
B_5_	Reliability and Data Security Concerns	Risks related to system reliability, cybersecurity, and data privacy due to the large volume of information generated and exchanged within the agri-food system.	
B_6_	High Cost of Facility Maintenance	High investment and operational costs required to establish and maintain the digital infrastructure and interoperable systems of Agriculture 4.0 in the agri-food system, including machinery, software, and telecommunications networks.	Economic
B_7_	High Cost of Skilled Labor	Elevated costs associated with hiring qualified professionals capable of operating, managing, and maintaining Agriculture 4.0 technologies within the agri-food system.	
B_8_	High Cost of Operational Components	High prices of essential technological components and decision-support solutions, such as advanced computing hardware, multispectral cameras, and specialized software used in the agri-food system.	
B_9_	Lack of Affordable Solutions for Farmers	Limited access to cost-effective technological solutions, as high investment requirements discourage farmers from adopting Agriculture 4.0 technologies in the agri-food system, such as autonomous machinery and agricultural robots.	
B_10_	Environmental, Ethical, and Social Concerns	Environmental, ethical, and social implications arising from the large-scale introduction of Agriculture 4.0 in the agri-food system, including impacts on natural preservation areas, energy use, and rural workers’ health.	
B_11_	Limited Availability and Accessibility	Insufficient availability and accessibility of Agriculture 4.0 technologies in the agri-food system, reinforcing the need for agricultural policies that stimulate and promote their adoption.	Political
B_12_	Lack of Farm- and Farmer-Centered Approaches	Absence of institutional and organizational structures—such as cooperatives, government agencies, and private enterprises—focused on farmers’ needs and farm-specific conditions within the agri-food system.	
B_13_	Lack of Clear Action Plans for Technology Implementation	Absence of structured strategies and public action plans to guide and facilitate the implementation of Agriculture 4.0 technologies across the agri-food system.	
B_14_	Regulatory and Data Governance Challenges	Lack of updated regulations, procedures, and agreements related to land ownership, data use, privacy, and the operation of autonomous agricultural machinery within the agri-food system.	
B_15_	Insufficient R&D and Innovative Business Models	Weak integration between universities, research institutions, and innovation ecosystems, combined with limited investment in R&D and scalable business models to support Agriculture 4.0 in the agri-food system.	
B_16_	Educational Gaps	Inadequate agricultural education systems that fail to incorporate the competencies required for Agriculture 4.0, such as digital skills, data analysis, and technology management within the agri-food system.	Social
B_17_	Age-Related Adoption Risk	Low adoption of Agriculture 4.0 technologies among older farmers and other actors in the agri-food system.	
B_18_	Lack of Digital Skills	Insufficient digital and technological skills among actors in the agri-food system, limiting the effective implementation and use of Agriculture 4.0 technologies.	
B_19_	Information Asymmetry	Lack of clear guidelines and effective communication, resulting in limited understanding of the benefits and practical applications of Agriculture 4.0 across the agri-food system.	
B_20_	Disruption of Existing Work Practices	Interruptions and operational challenges caused by the introduction of new digital technologies into established workflows within the agri-food system.	
B_21_	Climate and System Behavior Challenges	Adverse environmental conditions that reduce the durability, performance, and life cycle of Agriculture 4.0 technologies operating in the agri-food system.	Environmental
B_22_	Low Effectiveness of Rural Data	Limitations in the accuracy and reliability of environmental and climate data collected in rural contexts of the agri-food system, such as temperature, humidity, soil moisture, solar radiation, and precipitation.	
B_23_	Sustainability Constraints	Restrictions related to changes in food production, consumption patterns, and food waste management practices required by Agriculture 4.0 within the agri-food system.	
B_24_	Limited Farm-Level Data Collection Techniques	Challenges in developing efficient and reliable techniques for collecting high-quality data directly from farms operating within the agri-food system.	
B_25_	Limitations in Sustainable Energy Supply	Dependence on sustainable energy sources that, although environmentally appropriate, may present low productivity or limited availability for Agriculture 4.0 technologies in the agri-food system.	

**Note:** The barriers presented in this table were originally identified through a systematic literature review on Agriculture 4.0 and agri-food systems (see [5]). These barriers were subsequently validated in an independent empirical study (see [18]), ensuring their robustness and applicability within the agri-food system context.

**Table 2:** Cronbach’s Alpha Values for Agriculture 4.0 Barrier Clusters.

Barrier Cluster	Cronbach’s Alpha (α)	Interpretation
Technological	0.828	Strong internal consistency
Economic	0.744	Acceptable internal consistency
Political	0.783	Acceptable to strong consistency
Social	0.829	Strong internal consistency
Environmental	0.673	Marginally acceptable internal consistency

**Table 3:** Distribution and qualitative interpretation of clusters based on PCA-reduced barrier perceptions.

Cluster	Number of Observations	Description
Cluster 0	39	Respondents are located near the centroid of the principal component space, characterized by moderate and relatively balanced barrier perceptions. This group does not express strongly polarized views, suggesting a neutral or transitional stance toward Agriculture 4.0 adoption.
Cluster 1	9	Respondents with strongly negative PC1 scores and varying PC2 values, predominantly concentrated in the lower-left quadrant of the component space. This cluster reflects a more skeptical group, likely experiencing higher perceived constraints related to core adoption barriers, limited infrastructure, or restricted access to enabling conditions.
Cluster 2	12	Respondents exhibited negative PC2 scores across a range of PC1 values, indicating comparatively moderate perceptions of primary barriers but heightened concern with secondary dimensions such as education, digital skills, institutional learning processes, and socio-environmental considerations.

## Data Availability

The data presented in this study are available on request from the corresponding author.

## Appendix A

Table A1 presents the questionnaire used as the data collection instrument in this study, providing a general overview of the structure, sections, and main themes addressed in the survey.

**Table A1:** Questionnaire Applied for Data Collection.

Barriers to the Adoption of Agriculture 4.0 Technologies in Rio Grande do Sul—Brazil
PART I—Questionnaire Introduction
Dear farmer, The purpose of this questionnaire is to analyze farmers’ perceptions of a set of barriers that hinder the adoption of digital technologies associated with Agriculture 4.0 in the agri-food system of Rio Grande do Sul (RS), Brazil. Participation in this survey is voluntary. All responses will be used exclusively for scientific purposes, ensuring full anonymity and confidentiality. Data will be analyzed in aggregated form, with no individual identification of respondents. The estimated time to complete the questionnaire is 5 to 10 min. Thank you for your collaboration.
**PART II—Respondent Characterization (Demographic Questions)**
**1.** Gender:
( ) Male ( ) Female
**2.** Age (in completed years):
**3.** Highest level of education completed: ( ) Elementary education ( ) High school ( ) Technical high school ( ) Undergraduate degree ( ) Master’s degree ( ) Doctoral degree
**4.** City where the main farm/property is located:
**5.** Total agricultural area used for crop production: ( ) Up to 20 hectares ( ) From 21 to 100 hectares ( ) More than 100 hectares
**6.** Main type of agricultural crop produced: ( ) Soybean ( ) Wheat ( ) Corn ( ) Rice ( ) Oats ( ) Tobacco ( ) Fruit production (e.g., grapes, apples) ( ) Other:
**7.** Length of experience with this crop (in years):
**8.** On a scale from 1 to 5, how would you rate your level of knowledge about Agriculture 4.0? (1 = Do not understand at all; 5 = Understand very well). *Note: Agriculture 4.0 refers to the implementation of emerging technologies (such as drones, big data, cloud computing, autonomous mobile robots, and smart sensors) within the agri-food system. These technologies support activities across the agri-food value chain—from agricultural production to processing, logistics, and distribution—and require cultural and organizational changes to improve productivity and efficiency through real-time, data-driven decision-making.* ( ) 1—Do not understand ( ) 2—Understand a little ( ) 3—Understand moderately ( ) 4—Understand ( ) 5—Understand very well
**PART III—Barriers to the Adoption of Agriculture 4.0 Technologies**
For each statement below, please indicate the level of importance of the barrier in your decision to adopt Agriculture 4.0 technologies, using the following scale: (1) Not important (2) Slightly important (3) Moderately important (4) Important (5) Very important
**TECHNOLOGICAL BARRIERS**
**Technological Complexity—**Difficulties arising from the low usability of technological equipment (e.g., autonomous machines, sensors, apps, and software), due to insufficient training, poor ergonomics, or inadequate user interfaces. ( ) 1 ( ) 2 ( ) 3 ( ) 4 ( ) 5
**Incompatibility between Components—**Constraints related to integrating equipment and software from different providers with existing operations, including limited interoperability among sensors and platforms. ( ) 1 ( ) 2 ( ) 3 ( ) 4 ( ) 5
**Energy Management Problems—**Limitations associated with energy availability and power consumption, especially battery life and autonomy of drones and autonomous robots. ( ) 1 ( ) 2 ( ) 3 ( ) 4 ( ) 5
**Lack of Infrastructure—**Insufficient telecommunications and digital infrastructure on farms, limiting rural connectivity. ( ) 1 ( ) 2 ( ) 3 ( ) 4 ( ) 5
**Reliability and Data Security Concerns—**Risks related to system reliability, cybersecurity, and data privacy due to large volumes of data. ( ) 1 ( ) 2 ( ) 3 ( ) 4 ( ) 5
**ECONOMIC BARRIERS**
**High Cost of Facility Maintenance—**High investment and operational costs to establish and maintain digital infrastructure and interoperable systems. ( ) 1 ( ) 2 ( ) 3 ( ) 4 ( ) 5
**High Cost of Skilled Labor—**Elevated costs associated with hiring qualified professionals to operate and maintain technologies. ( ) 1 ( ) 2 ( ) 3 ( ) 4 ( ) 5
**High Cost of Operational Components—**High prices of essential components and decision-support solutions (e.g., advanced hardware, multispectral cameras, software). ( ) 1 ( ) 2 ( ) 3 ( ) 4 ( ) 5
**Lack of Affordable Solutions for Farmers—**Limited access to cost-effective solutions due to high investment requirements. ( ) 1 ( ) 2 ( ) 3 ( ) 4 ( ) 5
**Environmental, Ethical, and Social Concerns—**Implications of large-scale adoption, including impacts on preservation areas, energy use, and workers’ health. ( ) 1 ( ) 2 ( ) 3 ( ) 4 ( ) 5
**POLITICAL BARRIERS**
**Limited Availability and Accessibility—**Insufficient availability and accessibility of technologies, requiring supportive public policies. ( ) 1 ( ) 2 ( ) 3 ( ) 4 ( ) 5
**Lack of Farm- and Farmer-Centered Approaches—**Absence of institutional and organizational structures focused on farmers’ needs. ( ) 1 ( ) 2 ( ) 3 ( ) 4 ( ) 5
**Lack of Clear Action Plans for Technology Implementation—**Absence of structured strategies and public action plans to guide implementation. ( ) 1 ( ) 2 ( ) 3 ( ) 4 ( ) 5
**Regulatory and Data Governance Challenges—**Lack of updated regulations related to land ownership, data use, privacy, and autonomous machinery. ( ) 1 ( ) 2 ( ) 3 ( ) 4 ( ) 5
**Insufficient R&D and Innovative Business Models—**Limited integration between research institutions and innovation ecosystems and low R&D investment. ( ) 1 ( ) 2 ( ) 3 ( ) 4 ( ) 5
**SOCIAL BARRIERS**
**Educational Gaps—**Inadequate education systems that fail to develop Agriculture 4.0 competencies. ( ) 1 ( ) 2 ( ) 3 ( ) 4 ( ) 5
**Age-Related Adoption Risk—**Lower adoption rates among older farmers. ( ) 1 ( ) 2 ( ) 3 ( ) 4 ( ) 5
**Lack of Digital Skills—**Insufficient digital and technological skills limiting effective use. ( ) 1 ( ) 2 ( ) 3 ( ) 4 ( ) 5
**Information Asymmetry—**Limited understanding of benefits and applications due to poor communication. ( ) 1 ( ) 2 ( ) 3 ( ) 4 ( ) 5
**Disruption of Existing Work Practices—**Operational disruptions caused by new digital technologies. ( ) 1 ( ) 2 ( ) 3 ( ) 4 ( ) 5
**ENVIRONMENTAL BARRIERS**
**Climate and System Behavior Challenges—**Adverse environmental conditions reducing durability and performance. ( ) 1 ( ) 2 ( ) 3 ( ) 4 ( ) 5
**Low Effectiveness of Rural Data—**Limited accuracy and reliability of environmental and climate data. ( ) 1 ( ) 2 ( ) 3 ( ) 4 ( ) 5
**Sustainability Constraints—**Restrictions related to changes in production, consumption, and waste management. ( ) 1 ( ) 2 ( ) 3 ( ) 4 ( ) 5
**Limited Farm-Level Data Collection Techniques—**Challenges in collecting high-quality data directly from farms. ( ) 1 ( ) 2 ( ) 3 ( ) 4 ( ) 5
**Limitations in Sustainable Energy Supply—**Dependence on sustainable energy sources with limited productivity or availability. ( ) 1 ( ) 2 ( ) 3 ( ) 4 ( ) 5

**Note:** This questionnaire provides an overview of the data collection instrument used in this study. The barriers to the adoption of Agriculture 4.0 technologies in the agri-food system were assessed using a five-point Likert scale, in which higher values indicate greater perceived importance. Full methodological details regarding the development and application of the instrument are presented in the main text.
